# The importance of inter-individual Kupffer cell variability in the governance of hepatic toxicity in a 3D primary human liver microtissue model

**DOI:** 10.1038/s41598-019-43870-8

**Published:** 2019-05-13

**Authors:** Ali Kermanizadeh, David M. Brown, Wolfgang Moritz, Vicki Stone

**Affiliations:** 1 Heriot Watt University, School of Engineering and Physical Sciences, Nano Safety Research Group, Edinburgh, UK; 2 InSphero AG, Wagistrasse 27, Schlieren, Switzerland

**Keywords:** Risk factors, Hepatotoxicity

## Abstract

The potential for nanomaterial (NM) translocation to secondary organs is a realistic prospect, with the liver one of the most important target organs. Traditional *in vitro* or *ex vivo* hepatic toxicology models are often limiting and/or troublesome (i.e. short life-span reduced metabolic activity, lacking important cell populations, high inter-individual variability, etc.). Building on previous work, this study utilises a 3D human liver microtissue (MT) model (MT composed of mono-culture of hepatocytes or two different co-culture MT systems with non-parenchymal cell (NPC) fraction sourced from different donors) to investigate the importance of inter-donor variability of the non-parenchymal cell population in the overall governance of toxicological response following exposure to a panel of NMs. To the best of our knowledge, this is the first study of its kind to investigate inter-donor variability in hepatic NPC population. The data showed that the Kupffer cells were crucial in dictating the overall hepatic toxicity following exposure to the materials. Furthermore, a statistically significant difference was noted between the two co-culture MT models. However, the trend for particle-induced biological responses was similar between the co-cultures (cytotoxicity, cytokine production and caspase activity). Therefore, despite the recognition of some discrepancies in the absolute values between the co-culture models, the fact that the trends and patterns of biological responses were comparable between the multi-cellular models we propose the 3D liver MT to be a valuable tool in particle toxicology.

## Introduction

The prompt expansion, exploitation and commercial use of engineered nanomaterials (NMs) has resulted to considerable interest in the fields of nanomedicine and nanotechnology^1,2^. Unfortunately, the same unique physicochemical characteristics (small size, charge, shape, solubility etc.) which make NMs sought-after might also contribute to their toxicity and conceivable adverse effects. With the inevitable rise for public and occupational exposure from increasing production and utilisation of NMs, there is an imperative need to consider the potential detrimental health consequences of material exposure^3–5^.

The lungs and the gastrointestinal tract are are the primary exposure sites for NMs^3,6^. However, it is well established that a proportion of NMs can translocate to a range of secondary sites with the liver being one of the most important (the organ has been demonstrated to be an important accumulation site for materials as compared to others)^4,7–10^. Furthermore, the constant enhancement in the development of nanomedicines will result in direct entry of NMs into the bloodstream. This will result in the materials reaching the liver rapidly and in large quantities^4,7^.

The liver is the bodies’ main detoxification centre, removing xenobiotics and waste products^11^. The organ is composed of distinct populations of cells, amongst which the hepatocytes (parenchymal cells), the resident macrophage population (Kupffer cells - KCs) and sinusoidal endothelial cells are amongst some of the most important^12–14^. In particular and of huge significance in particle hepatotoxicity is the fact that the KCs line the liver sinusoids. This locality means that these cells have constant contact with gut-derived antigens as well as any material, which might reach the organ from the bloodstream. Additionally, once activated KCs are amongst the most important cell populations in the modulation and governance of overall hepatic immune response in the non-diseased organ^14,15^.

Based on the progression in the field of nanotoxicology over the last decade, it would be reasonable to state that in all reality, any potential NM-induced adverse effects in the liver would only occur after long-term chronic exposure in man. Therefore, it is essential to establish more advanced, physiologically relevant *in vitro* assessment tools for improved prediction of the adverse effects caused by chronic NM exposure in humans. The utilisation of human primary hepatic cells is the closest representative *in vitro* model for the human liver. However, these cells are phenotypically unstable in 2D cultures and have a very limited life-span (typically no longer than 7 days - with continued reduced viability, functional and metabolic activity). In addition, there is large variability between primary cells sourced from different donors^16–18^. Moreover, in most traditional 2D hepatic models, non-parenchymal cell (NPC) populations are not included or considered.

The aforementioned inter-individual variability is one of the prevalent drawbacks of the use of primary cells for metabolic or toxicological studies (in particular immune cells^19–21^). Understanding the patterns and the nature of biological responses from the immune cells (KCs in the liver) sourced from different individuals is critical for good experimental design and more accurate *in vitro* to *in vivo* data extrapolation.

In an attempt to address this issue, this study was designed to scrutinize a scaffold free 3D liver microtissue (MT) model composed of primary human hepatocytes and primary human liver-derived NPC (particular attention on inter-individual variability in the NPC population). To best of our knowledge, this is the first study, to investigate the importance of the role of KCs in the particle-induced hepatic inflammatory response *in vitro*, as well ascertaining the significance of inter-donor NPC sub-population variability in the toxicological response from the liver. In this study, three different MT models were utilized: (1) MT composed of mono-culture of hepatocytes (pooled from 10 different donors) (2) co-culture MT composed of multi-donor primary human hepatocytes and NPC fraction from donor 1, while (3) co-culture MT is composed of multi-donor primary human hepatocytes and NPC fraction from donor 2. Increased knowledge of the biological capabilities of the test model systems will no doubt be critical for better understanding the outcome of experiments intended to evaluate liver-specific function and toxicity and prove invaluable in future study design.

In these experiments, the MT were exposed to a panel of four NMs with different physicochemical properties - namely - a multi-walled carbon nanotube (MWCNT), silver (Ag), titanium dioxide (TiO_2_) and zinc oxide (ZnO) (all Tier 1 NMs in H2020 funded PATROLS project^22^). The materials are representative of NMs currently incorporated in various consumer products including sunscreens, cosmetics, clothing and sporting goods^2^. The toxicological effects of the NMs was assessed on the different human liver MT following a singular or repeated exposures for up to 7 days. It is important to clearly state that the focus of this study is predominately on the three different MT models and not on the toxicological profiling of the NMs.

## Material and Methods

### Liver MT maintenance

In these experiments a total of thirty 96 well MT plates were utilized: (**1)** 10 × 3D InSight^™^ multi-donor human liver MT containing primary hepatocytes in mono-culture (hepatocyte lot IPHH_11 (pooled cells from 10 donors)) (InSphero AG, Switzerland); (**2)** 10 × 3D InSight^™^ multi-donor human liver MT composed of multi-donor primary human hepatocytes in co-culture with a single donor NPC containing primary Kupffer cells and primary liver endothelial cells (hepatocyte lot IPHH_11, Kupffer cell lot IPHN_12) (InSphero AG, Switzerland) (termed co-culture 1) and (**3)** 10 × 3D InSight^™^ multi-donor human liver MT composed of multi-donor primary human hepatocytes in co-culture with a single donor NPC (hepatocyte lot IPHH_11, Kupffer cell lot IPHN_08) (termed co-culture 2) (InSphero AG, Switzerland) (Table 1). The InSphero MTs is a commercial product with the company sourcing cryopreserved primary cells from different suppliers, where “Ethics Policy Statements” provided by the primary cell supplier confirm adherence to ethical standards (no additional institutional approval was required for these experiments).

**Table 1:** Human NPC donor demographics and characterization.

Donor	1	2
InSphero lot no	IPHN_12	IPHN_08
Ethnicity	Caucasian	Hispanic
Gender	Female	Male
Age	51	44
Cause of death	Natural cause - ventricular fibrillation	Anoxia
Smoker	Unknown	Smoked one pack of cigarettes per day between the age of 15–29
Drugs	Unknown	Marijuana and cocaine use in the past
Epstein-Barr virus	Unknown	Unknown
Cytomegalovirus	Negative	Positive
Hepatitis B	Negative	Negative
Hepatitis C	Negative	Negative
HIV	Negative	Negative

The human liver MT (both mono-culture and co-cultures) was maintained in complete medium (3D InSight^™^ human liver maintenance medium - AF (InSphero AG, Switzerland)) at 37 °C, 5% CO_2_, 95% humidity with the medium exchanged (70 µl per well) on the day of arrival and every 2 days thereafter (50 µl per well).

It is important to state that the MT have been thoroughly characterised (histology, albumin production and basal and inducible cytochrome P450 activity) for up to 5 weeks in culture; with the data reported elsewhere^23^.

### Nanomaterials

The NMs were sourced as follows: TiO_2_ (JRC Nanomaterials Repository - Italy, JRCNM01005a), ZnO (JRC Nanomaterials Repository - Italy, JRCNM01101a), Ag (Fraunhofer IME - Germany, NM300-K) and MWCNT (National Research Centre for Working Environment - Denmark, MITSUI-7). The NMs were sub-sampled under Good Laboratory Practice conditions and preserved under argon in the dark until use (with the exception of the MWCNT).

### Characterisation of the panel of NMs

A summarised list of the measured physical and chemical properties of the selected NMs has been re-produced from previous work^24–26^ (Table 2). Furthermore, the hydrodynamic size distributions of the NMs dispersed in complete medium were determined at a concentration of 25 µg/ml by Dynamic Light Scattering (DLS) using a Zetasizer Nano-ZS (Malvern, USA) (Table 2). The employed instrument can recognize particles in the 0.5–6000 nm range. Finally, a Pierce LAL Chromogenic Endotoxin Quantitation Kit (Thermo Scientific, UK) was utilised to test for possible endotoxin contaminations of the tested NMs. The kit was used according to the manufacturer’s guidelines.

**Table 2:** Main physical and chemical properties of investigated NMs (adapted and reproduced from^24–26^).

NM code	NM type	Phase	Primary size (nm)	Surface area [m^2^/g] (BET)	Known coating	Size in liver maintenance medium (DLS) (nm)^Ψ^
JRCNM01005a	TiO_2_	Rutile-anatase	15–24	46	None	165 ± 5.2
JRCNM01101a	ZnO	Coated	152	15	Triethoxycaprl-silane	196 ± 4.4
NM300-K	Ag	—	15	—	4% each of polyoxyethylene glycerol trioleate and Tween 20	59.9 ± 1.9
MITSUI-7	MWCNT	—	D - 40–50 L - 13 µm	—	None	875.4 ± 94.5

^Ψ^Size in biological media measured within 30 min of sonication.

### NM dispersion and treatment

NMs were prepared following the NANOGENOTOX dispersion protocol^27^. Following the sonication step, all materials were immediately transferred to ice before being diluted in complete medium just prior to the experiments. In this experiment, five NM concentrations were used: 1.5, 3, 6, 12 and 25 µg/ml (total volume of 50 µl added to each well) as well as negative (cell culture medium) and positive (1% Triton-X or Camptothecin) (Sigma, UK) controls. The first NM exposure took place 48 hr after the MT was received. For each treatment, five MT were used and all experiments were repeated on three separate occasions (15 wells − 5 wells for each material concentration or control on three occasions). The material treatment regimes in the singular and repeated exposure experiments are summarised in Table 3. To avoid potential complications with aging of tissue between different plates, the three NM treatment repetitions were carried on the same day (morning, lunchtime and late afternoon - with a fresh NM batch prepared for each exposure as described above). Importantly, all concentrations are expressed as µg/ml. The principal reason for this is that the liver cells are clustered in spheroids and expressing the doses as µg/cm^2^ would not be appropriate.

**Table 3:** NM treatment in the singular and repeated exposure experiments over a period of 7 days.

Single exposure	End-points investigated
Material Treatment at 0 hr	—
Medium removal and tissue harvest at **24 hr**	AK assay, cytokine secretion, albumin, caspase activity
**Repeated exposure**	**End-points investigated**
Material Treatment **1** at 0 hr	—
Medium removal at 24 hr 24 hr recovery period	AK assay, cytokine secretion, albumin
Material Treatment **2** at 48 hr	—
Medium removal at **72 hr** 24 hr recovery period	AK assay, cytokine secretion, albumin
Material Treatment **3** at 96 hr	—
Medium removal and tissue harvest at **120 hr**	AK assay, cytokine secretion, albumin, caspase activity

### Adenylate kinase (AK) assay

The loss of cell membrane integrity was evaluated utilising a ToxiLight™ bioassay kit (Lonza, USA). Briefly, 20 µl of cell supernatant was transferred to a luminescence compatible plate before the addition of 80 µl of AK detection buffer. The plates were incubated for 5 min at room temperature and luminescence measured. Triton-X was utilised as a positive control in the cytotoxicity assay (24 hr exposure)^28^.

### Cytokine secretion

The levels of human interleukin (IL)6, IL8 and IL10 secreted from the MT was determined in the cell supernatant using R&D Systems magnetic Luminex^®^ Performance Assay multiplex kits (bead based immunoassay; Bio-techne, USA) according to the manufacturers instruction. The proteins were detected via a Bio-Rad^®^ Bio-Plex^®^ MAGPIX multiplex reader. The technology is constructed on the use of analyte-specific antibodies pre-coated onto magnetic microplates embedded with fluorophores at set ratios for each unique microparticle region.

### Albumin production

After exposure the supernatants (from both the control and treated cells as described above) were collected and stored at −80 °C. The supernatants were centrifuged at 1000 g, diluted two fold and albumin levels determined by ELISA according to the manufacturer’s instructions (Bethyl laboratories, USA)^28^.

### Caspase activity assay

Caspase-Glo^®^ 3/7 reagents (Promga, UK) were prepared according to manufacturer’s instructions. For these experiment 6 µM of Camptothecin (inducer of apoptosis) was used as positive control (24 hr exposure). Following the exposures, the MT were removed from the incubator and allowed to equilibrate to room temperature. At this juncture, 50 µl of Caspase-Glo^®^ 3/7 reagent was added to all wells before incubation at room temperature for 90 min. The well contents were transferred to a luminescence compatible plate and measured using a luminometer.

### Statistical analysis

All data are expressed as mean ± standard error of mean (SEM). For statistical analysis, the experimental results were compared to their corresponding control values using full-factorial ANOVA with Tukey’s multiple comparison. All statistical analysis was carried out utilizing Minitab 18. A p value of < 0.05 was considered to be significant. The experiments were repeated on a minimum of three separate occasions (n = 3).

## Results

### Characteristics of pristine and dispersed NMs

The NMs utilised in this study were thoroughly characterised by a permutation of analytical techniques which have been described previously^24–26^ and reproduced in Table 2. Furthermore, to investigate how the NMs behaved in the liver maintenance medium, the hydrodynamic size distribution of the materials was investigated (Table 2). In addition, no endotoxin contamination (≤0.25 EU/ml) was detected in any of the NM suspensions.

### Impact of NM exposure on MT cell membrane integrity

**Figure 1:**
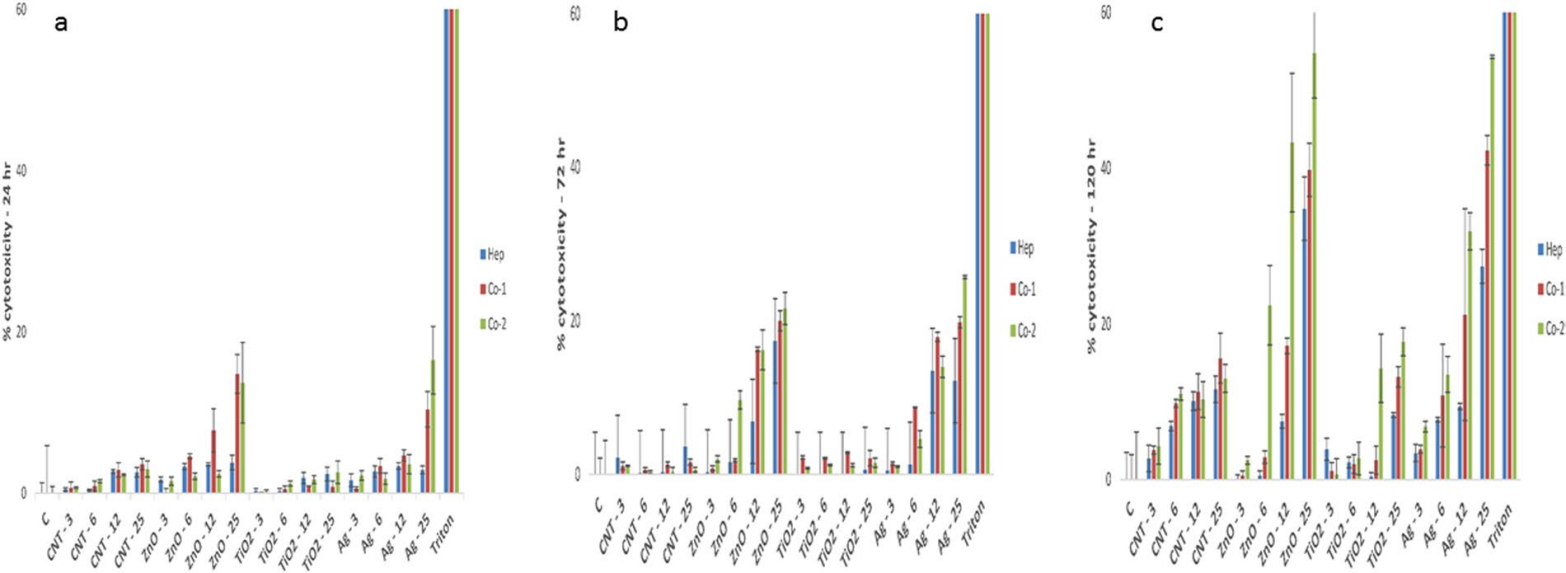
Cytotoxicity in human liver MT (mono-culture of hepatocytes only, co-culture of hepatocytes with NPC fraction from donor 1 and co-culture of hepatocytes with NPC from donor 2) following single or repeated exposures to a panel of engineered NMs for up 120 hr. The cytotoxicity as measured by AK release via ToxiLight™ cytotoxicity assay (**a**) 24 hr (**b**) 72 hr and (**c**) 120 hr (NM treatments µg/ml). Triton-X was utilised as a positive control in the cytotoxicity assay (24 hr exposure). The values represent mean ± SEM (n = 3) with significance indicated by *p < 0.05 and **p < 0.005 of NM-induced effects compared to negative control, ^#^p < 0.05 and ^##^p < 0.005 signifying differences between the mono-culture and the co-culture and ^$^p < 0.05 and ^$$^p < 0.005 highlighting variation between co-culture 1 and co-culture 2.

The AK data showed a concentration and time dependent decrease in cell membrane integrity following repeated exposure to the NMs (Fig. 1). These effects were more evident after exposure to the ZnO and Ag NMs at 72 and 120 hr (Fig. 1b,c) (p < 0.05). Importantly, a LC_50_ was not reached for any of the NMs at any of the time-points or concentrations investigated. Interestingly and crucially, the general NM toxicity profile over time was greater for the two co-cultures as compared to the hepatocyte only MT. This increase in toxicity could potentially be explained by enhanced uptake of NMs by the phagocytes in the co-cultures. Furthermore, the NM-induced cell death was more notable in the co-culture 2 (KC from donor 2) as compared to co-culture 1 (KC from donor 1). Importantly, no NM interference was noted with this assay^35^.

### Cytokine secretion from liver MT following NM exposure

Next, the changes in cytokine production levels (IL6, IL8 and IL10) following NM exposure was assessed within the supernatant of the material treated liver MT. IL6 is an important mediator of innate immunity. The protein is secreted by macrophages; and instigates various innate immune signalling cascades resulting in an augmentation of inflammatory responses and cell recruitment. IL6 is also essential for liver regeneration^29^. Furthermore, IL6 is responsible for stimulating acute phase protein response form the liver as well as the production of neutrophils in the bone marrow^30,31^. IL8 is a potent chemokine, important in the activation and migration of a wide variety of inflammatory cells governing innate immunity, hence crucial in the initiation of an inflammatory response^32,33^. IL10 is a multi-functional cytokine with a wide and diverse range of effects. The cytokine’s main function is to terminate inflammatory responses^34,35^. The cytokine plays a crucial role in the differentiation of regulatory T lymphocytes, involved in the control of inflammatory responses and development of an immune tolerance^35^.

**Figure 2:**
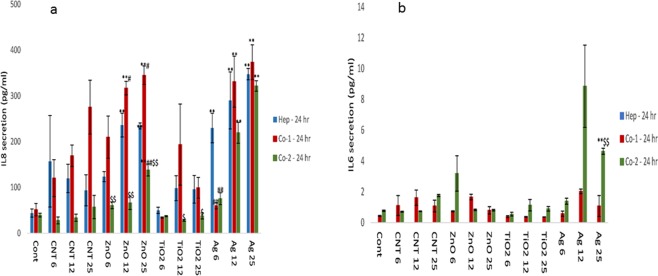
IL8, IL6 secretion from NM exposed human liver MT. The tissues were exposed to cell medium (cont) or NMs for a period of 24 hr (**a)** IL8 (**b)** IL6. The values represent mean ± SEM (n = 3) with significance indicated by *p < 0.05 and **p < 0.005 of NM-induced effects compared to negative control, ^#^p < 0.05 and ^##^p < 0.005 signifying statistical differences between the mono-culture and the co-culture and ^$^p < 0.05 and ^$$^p < 0.005 highlighting variation between co-culture 1 and co-culture 2.

**Figure 3:**
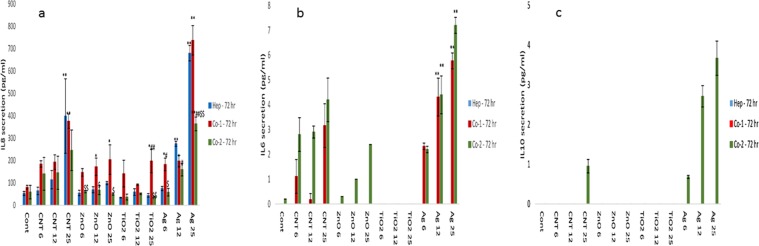
IL8, IL6 and IL10 secretion from NM exposed human liver MT. The tissues were exposed to cell medium (cont) or NMs for a period of 72 hr (**a)** IL8 (**b)** IL6 and (**c)** IL10. The values represent mean ± SEM (n = 3) with significance indicated by *p < 0.05 and **p < 0.005 of NM-induced effects compared to negative control, ^#^p < 0.05 and ^##^p < 0.005 signifying statistical differences between the mono-culture and the co-culture and ^$^p < 0.05 and ^$$^p < 0.005 highlighting variation between co-culture 1 and co-culture 2.

**Figure 4:**
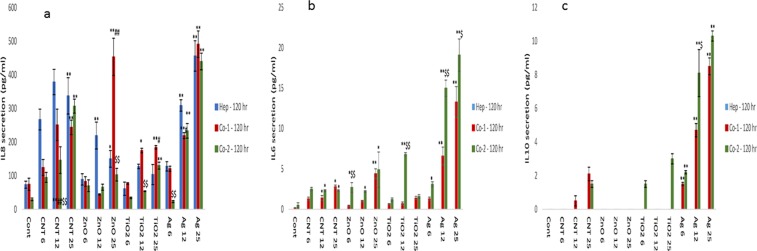
IL8, IL6 and IL10 secretion from NM exposed human liver MT. The tissues were exposed to cell medium (cont) or NMs for a period of 120 hr (**a)** IL8 (**b)** IL6 and (**c)** IL10. The values represent mean ± SEM (n = 3) with significance indicated by *p < 0.05 and **p < 0.005 of NM-induced effects compared to negative control, ^#^p < 0.05 and ^##^p < 0.005 signifying statistical differences between the mono-culture and the co-culture and ^$^p < 0.05 and ^$$^p < 0.005 highlighting variation between co-culture 1 and co-culture 2.

For the single treatment experiments, an increase in IL8 was observed for three of the four NMs in a concentration-dependent fashion (with the exception of TiO_2_ NMs). No discernible pattern was notable between the mono-culture and the co-culture MT (although a number of statistically significant differences were recorded i.e. Ag NM − 6 µg/ml) (Fig. 2a). Unsurprisingly, IL6 secretion was only observed in the co-cultures and most evident for the Ag NM at 24 hr exposure (Fig. 2b). Moreover, no IL10 secretion was detected from either the mono-culture or co-culture MT at 24 hr (data not shown). At 72 and 120 hr, a similar a pattern was observed for the IL8 and IL6 secretion from the MT (albeit at higher concentrations compared to 24 hr exposure) (Figs 3a,b, 4a,b). Interestingly, at 72 and 120 hr there was an additional IL10 response (Figs 3c and 4c), which was most significant following exposure to the Ag NMs (Fig. 3c (p < 0.005) and 4c (p < 0.005)). It is important to state that IL6 and IL10 release profiles was generally higher for co-culture from NPC fraction donor 2 compared to donor 1 (similar pattern as observed for the cytotoxicity data). Finally, it is worth mentioning that the NM-induced IL10 secretion levels in this set of experiments was considerably lower than our previously published work^36^. Some of the potential reasons for these differences are discussed in upcoming sections.

### Albumin production by human liver MT

Next, NM-induced effects on albumin production (as a marker of hepatocyte function) was investigated at 24 hr or 120 hr, with a low and high concentration (3 and 25 µg/ml) selected for each NM. The data showed that there were no significant changes at either of the investigated concentrations or time-points following exposure of four NMs. Furthermore, no significant difference between albumin levels was noted between the mono-culture and co-culture MT (data not shown).

### Caspase activity in the NM exposed MT

**Figure 5:**
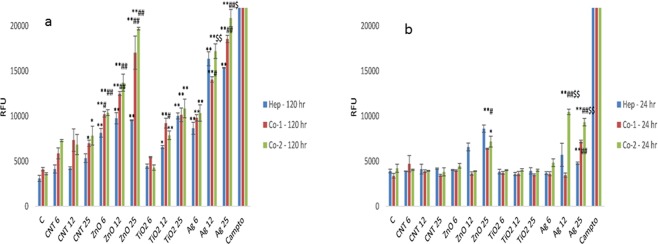
Caspase activity in 3D human liver MT. The effect of NM exposure (or positive control Camptothecin (Campto)) on caspase 3/7 activity in the human liver MT (**a)** 24 hr (**b)** 120 hr. The values represent mean ± SEM (n = 3) with significance indicated by *p < 0.05 and **p < 0.005 of NM-induced effects compared to negative control, ^#^p < 0.05 and ^##^p < 0.005 signifying statistical differences between the mono-culture and the co-culture and ^$^p < 0.05 and ^$$^p < 0.005 highlighting variation between co-culture 1 and co-culture 2.

Caspase 3/7 activity was monitored in the NM-exposed MT or the controls at 24 hr and 120 hr (Fig. 5). As expected caspase activity was greater at 120 hr compared to 24 hr where NM-induced cell death was greater (most noticeable for the Ag and ZnO NMs). Overall, this data set showed very good correlation with the cytotoxicity results suggesting that apoptosis might be one of the mechanism of NM-induced cell death. This is not surprising, as we have previously shown that the Ag and ZnO NMs caused a dysfunction in the autophagy pathway; which proceeded apoptotic cell death in a hepatic cell line^37^. However, it is very interesting to note that the caspase activity was significantly higher in the NM exposed the two co-culture MT compared to the hepatocyte only model. This observation indicates at a significantly increased interaction of the KCs with the NMs and subsequent cell death in this sub-population. These findings clearly highlight the importance of the incorporation of KCs in any hepatic model used for hazard assessment of particulates. Finally, a significant difference in caspase activity was observed between the two different co-cultures following a 24 hr exposure to the Ag NM. Very similar to the observations for the cell membrane integrity and cytokine production the co-culture with NPC faction form donor 2 had an increased response following the NM challenge. Although a similar pattern was noted at 120 hr; the differences between the two co-cultures was not statistically significant.

## Discussion

This study was designed to assess the importance of inter-donor variability in the NPC cell population in particle-induced hepatic toxicity in a 3D liver MT model. The MT offers a functionally active system in a 96 well plate format with no scaffolds or hydrogels, that utilises primary human hepatic cells. Additionally, the model allows for one MT per well with a defined cell number. The liver cells in this model are viable and metabolically active for long periods, which allows for low dose multiple particulate treatments over a period of weeks. This equates to more physiologically relevant and realistic exposure scenarios and might prove to be a suitable surrogate model for investigating hepatic toxicity and disease progression *in vivo*. In order to achieve our aims, 3 MT models (hepatocyte only mono-culture and two co-cultures incorporating NPC from different donors) were exposed to a panel of four NMs with different physicochemical characteristics (ZnO, Ag, MWCNT and TiO_2_ NMs). The toxicological effects of the panel of NMs was assessed on the human liver MT following a singular or repeated exposures for up to 5 days.

The data from this study showed that the KCs were crucial in dictating the overall hepatic toxicological response following NM exposure (most evident following multiple exposure of the highly toxic Ag and ZnO). Furthermore, a statistically significant difference was noted between the two co-culture MT models in terms of cytotoxicity, caspase activity and cytokine secretion (presumed to be increased interaction or uptake of the materials by the NPC population). Interestingly, a stronger toxicological response was observed for co-culture 2 as compared to co-culture 1 for all relevant end-points investigated. As an important note, the possibility of NM interference with all the investigated toxicological end-points in this study have been assessed as a crucial component of generating standard operating protocols for the PATROLS project.

As touched upon, the focus of this study was solely on the MT models. Therefore, the toxicity of the NMs and the specifics of the end-points investigated will not be discussed in detail. However, it is important to state that the data presented in this study is in good accordance with our previously published work on various hepatic models both *in vitro* and *in vivo* (hepatocyte cell lines, primary human hepatocytes, 3D liver MT and healthy and diseased livers in mice)^13,25,35,38–41^. We believe that the variances in the biological responses between the materials are due to the differences in the solubility of the NMs rather than size effects. Our previous studies have demonstrated that the highly soluble ZnO an Ag induce a stronger hepatic biological response as compared to the low solubility materials (TiO_2_ and MWCNT).

KCs (resident liver macrophages) are localized within the lumen of the sinusoids. Importantly, these cells adhere to the endothelial cells that constitute the blood vessels. KCs are the first immune cells in the liver that come in contact with the gut bacteria^42^, and any particulate transported to the liver via the portal vein. Under normal circumstances, KCs play a critical role in maintaining liver immune tolerance (these cells are in a permanent semi-activated state principally due to the continuous exposure to antigens reaching the organ from the gut). However, in pathological conditions, they can be activated and fully differentiate into M1 or M2-like macrophages plating a pivotal role in the induction and amplification of the immune response^43^. Due to their locality in the liver sinusoids (first and most important cell population that encounter non-soluble particulates reaching the organ), it is crucial that KCs are incorporated in next generation *in vitro* hepatic models for hazard assessment of particles.

The use of *in vitro* hepatocyte models have been beneficial for the last three decades in research. Conventionally, hepatocytes are regarded as the most important cell population for drugs and chemical toxicity screening. This is understandable as drugs and chemical toxicity is predominately governed by the metabolism of the xenobiotic (frequently the metabolic intermediate substances are hepatotoxic). However, since bio-persistent NMs are not necessarily metabolised by hepatocytes, but rather first interact and/are internalised by KCs^44,45^ the use of hepatocyte only mono-cultures is illogical for particle hepatic toxicity screening. It is not entirely inconceivable that in all reality only a minuscule proportion of the administered/accidental dose of any bio-persistent material would even reach the hepatocytes *in vivo*. This highlights that a good understanding of the physiology (i.e. locality of cell populations) of the organ of interest is just as important as other well-established parameters (physiochemical characteristics of the materials, dispersion, dose, route of exposure, protein corona, etc.) in a high-quality particle toxicology study.

To date, the utilisation of primary human hepatocytes are the closest representation of the human liver. However, cryopreserved hepatocytes are generally phenotypically unstable, and have a very limited life-span. Moreover, there are significant variations between different human donors^16,46,47^. Furthermore, almost all *in vivo* and most *in vitro* primary cell hepatic nanotoxiclogy data have been generated using rodent models/cells. This is far from ideal as inter-species differences could prove to be pivotal in time both in the fields of nano/toxicology and nanomedicine. To address the limitations of species and donor variability, the MT utilised in this study, were manufactured using pooled human primary hepatocytes from 10 donors. Importantly, in the manufacture of the MT only suitable donor lots were selected - this was only possible by having access to a large repository of cryopreserved human hepatocytes from commercial suppliers^23^.

Very similar to the hepatocytes, the inter-donor variability of primary immune systems is one of the prevalent drawbacks of *in vitro* experimentation (including the hepatic system). Unfortunately, the necessity for the sourcing and use of NPC from different individuals is completely unavoidable. Therefore, it is imperative that this inconsistency is investigated to establish the biological patterns and the nature of the responses. The information gathered will be extremely important in ascertaining the suitability of the use of test model in question as well as being crucial for good experimental design and more accurate *in vitro* to *in vivo* data extrapolation. Our findings in this study showed a statistically significant difference between the two co-culture MT models in terms of cytotoxicity, caspase activity and cytokine secretion. However, for most measurements (NMs, concentrations or time-points) the trend for particle-induced biological response was very similar (despite the differences the same overall conclusions were reached). Nonetheless, in most instances, a stronger material induced toxicological response was observed for co-culture 2 as compared to co-culture 1. One possible explanation for these differences could be the life-style of the donors from which the NPC populations were sourced. The IPHN_08 (donor 2) KCs originate from an individual with a history of alcohol and drug abuse. It is possible that the KCs from this donor were in a more activated state than the macrophages sourced from donor 1, who on paper had a healthier life-style (KC are in inherently tolerogenic state in a healthy liver)^47^. It should be also be stated that the levels of IL10 secreted from the two co-culture MT in this study was noticeably lower than those reported previously^36^. There are a number of potential explanations for these variances. Firstly, different multiplex systems were used to quantity cytokine secretion in the two experiments, hence different sensitivity and efficiency of the cytokine analysis kits could be a confounding factor. Additionally, the tested materials used in each study were not the same (although the NMs had roughly similar sizes). Finally, the NPC population was obtained from different donors in the two studies, which will have undoubtedly contributed to the disparity in the observed outcomes.

It is important to state that it is extremely difficult to make direct resemblances between *in vivo* and *in vitro* biological responses, and usually, *in vitro* findings can only be an indicator of what might occur *in vivo*. This can be explained by the fact that in all reality, biological responses between *in vitro* and *in vivo* system are rarely like for like; therefore, some caution is required in over-emphasizing the significance of a biological response *in vitro*. As an example, increased cytokine secretion by a cell population *in vitro* might not necessarily equate to an inflammatory response *in vivo*. Furthermore, it is almost impossible to ascertain actual cytokine concentrations that would cause a biological response *in vivo*. Moreover, biological responses are rarely limited to a single organ, and communication between different cells and organs is vital in a biological response to a xenobiotic challenge. Hence, the placement of too much emphasis on the absolute values *in vitro* systems might not be necessarily very meaningful. Therefore, despite the discussed discrepancies in the absolute values between the co-culture MT, the fact that the trends and patterns of biological responses were similar between the multi-cellular models suggests that the 3D liver MT model to be a valuable *in vitro* tool in particle toxicology. To the best of our knowledge, this is the first study to investigate inter-donor variability in the hepatic NPC population.

Despite the above-described advantages of the InSphero 3D human liver MT, there are also potential pitfalls/uncertainties, which need to be further investigated. Due to the tightly packed three-dimensional structure of the spheroids, there is some ambiguity whether the inner cells in the MT come in direct contact with bio-persistent particles. This issue is important as it could significantly influence the toxicological output. Moreover, visualisation of the interaction/uptake of materials by the different cell populations in the MT is an absolute necessity. These issues will be addressed in future experiments using the MT where it is aimed to use extremely low repeated dosing to better mimic realistic chronic exposure scenarios. These planned experiments will also incorporate physiologically relevant organ specific toxicological end-points to allow for direct and more meaningful comparisons between *in vitro* and *in vivo* data.

## References

[1] Kermanizadeh A, Powell L, Stone V, Møller P (2018). Nano delivery systems and stabilized solid drug nanoparticles for orally administered medicine - current landscape. International Journal of Nanomedicine.

[2] Vance ME (2015). Nanotechnology in the real world: Redeveloping the nanomaterial consumer products inventory. Beilstein Journal of Nanotechnology.

[3] Johnston H, Brown D, Kermanizadeh A, Gubbins E, Stone V (2012). Investigating the relationship between nanomaterial hazard and physicochemical properties: Informing the exploitation of nanomaterials with therapeutic and diagnosis applications. Journal of Controlled Release.

[4] Kermanizadeh A, Balharry D, Wallin H, Loft S, Møller P (2015). Nanomaterial translocation - the biokinetics, tissue accumulation, toxicity and fate of materials in secondary organs - a review. Critical Reviews of Toxicology.

[5] Tetley TD (2007). Health effects of nanomaterials. Biochemical Society Transactions.

[6] Sadauskas E (2009). Bio-distribution of gold nanoparticles in mouse lung following intratracheal instillation. Chemistry Central Journal.

[7] Balasubramanian SK (2010). Biodistribution of gold nanoparticles and gene expression changes in the liver and spleen after intravenous administration in rats. Biomaterials.

[8] Kermanizadeh A, Gaiser BK, Johnston H, Brown DM, Stone V (2014). Toxicological impact of engineered nanomaterials on the liver - a review. British Journal of Pharmacology.

[9] Lee JH (2013). Bio-persistence of silver nanoparticles in tissues from Sprague-Dawley rats. Particle and Fibre Toxicology.

[10] Lipka J (2010). Bio-distribution of PEG-Modified gold nanoparticles following intratracheal instillation and intravenous injection. Biomaterials.

[11] Kmiec Z (2001). Co-operation of liver cells in health and disease. Advances in Anatomy, Embryology and Cell Biology.

[12] Godoy P (2014). Recent advances in 2D and 3D *in vitro* Systems using primary hepatocytes, alternative hepatocyte sources and non-parenchymal liver cells and their use in investigating mechanisms of hepatotoxicity, cell signalling and ADME. Archives of Toxicology.

[13] Kermanizadeh A (2014). The role of Kupffer cells in the hepatic response to silver nanoparticles. Nanotoxicology.

[14] Teigs G, Lohse AW (2009). Immune tolerance: What is unique about the liver. Journal of Autoimmunity.

[15] Bottcher JP, Knolle PA, Stabenow D (2011). Mechanisms balancing tolerance and immunity in the liver. Digestive Diseases.

[16] den Braver-Sewradj SP (2016). Inter-donor variability of phase I/phase II metabolism of three reference drugs in cryopreserved primary human hepatocytes in suspension monolayer. Toxicology in Vitro.

[17] Josse R, Dumont J, Fautrel A, Robin MA, Guillouzo A (2012). Identification of early target genes of aflatoxin B1 in human hepatocytes, inter-individual variability and comparison with other genotoxic compounds. Toxicology and Applied Pharmacology.

[18] Liguori MJ, Blomme EA, Waring JF (2008). Trovafloxacin-induced gene expression changes in liver-derived *in vitro* systems: comparison of primary human hepatocytes to HepG2 cells. Drug Metabolism and Disposition.

[19] Bakiyeva LT, Brooks RA, Rushton N (2005). Inter-individual and intra-individual variability in TNF-alpha production by human peripheral blood cells *in vitro*. Cytokine.

[20] Hedl M, Abraham C (2012). IRF5 risk polymorphisms contribute of interindividual variance in pattern recognition receptor-mediated cytokine secretion in human monocyte-derived cells. Journal of Immunology.

[21] Maina V (2012). Bias in macrophage activation pattern influences non-alcoholic steatohepatitis (NASH) in mice. Clinical Science.

[22] PATROLS, https://www.patrols-h2020.eu/ - accessed 6 August (2018).

[23] Messner, S. *et al* Transcriptomic, proteomic, and functional long-term characterization of multicellular three-dimensional human liver microtissues. *Applied in Vitro Toxicology*, 10.1089/aivt.2017.0022 (2018). 10.1089/aivt.2017.0022 PMC7500040 32953943

[24] JRC nanomaterials repository, https://ec.europa.eu/jrc/sites/jrcsh/files/JRC-Nanomaterials-Repository-List-of-Representative-Nanomaterials.pdf - accessed 6 August (2018).

[25] Kermanizadeh A (2013). *In vitro* assessment of engineered nanomaterials using C3A cells: cytotoxicity, pro-inflammatory cytokines and function markers. Nanotoxicology.

[26] Poland CA (2008). Carbon nanotubes introduced into the abdominal cavity of mice show asbestos-like pathogenicity in a pilot study. Nature Nanotechnology.

[27] NANOGENOTOX, https://www.anses.fr/en/system/files/nanogenotox_deliverable_5.pdf accessed 12 May (2018).

[28] Kermanizadeh A (2014). Hepatic toxicology following single and multiple exposure of engineered nanomaterials utilising a novel primary human 3D liver microtissue model. Particle and Fibre Toxicology.

[29] Brock M (2011). MicroRNA-18a enhances the interleukin-6-mediated production of the acute phase proteins fibrinogen and heptoglobin in human hepatocytes. The Journal of Biological Chemistry.

[30] Tachibana S (2014). Interluekin-6 is required for cell cycle arrest and activation of DNA repair enzymes after partial hepatectomy in mice. Cell and Bioscience.

[31] Wong CP, Rinaldi NA, Ho E (2015). Zinc deficiency enhanced inflammatory response by increasing immune cell activation and inducing IL6 promoter demethylation. Molecular Nutrition and Food Research.

[32] Puthothu B, Krueger M, Heinze J, Forster J, Heinzmann A (2006). Impact of IL8 and IL8-receptor alpha polymorphisms on the genetics of bronchial asthma and severe RSV infections. Clinical and Molecular Allergy.

[33] Takahashi A, de Andres MC, Hashimoto K, Itoi E, Oreffo RO (2015). Epigenetic regulation of interleukin-8, an inflammatory chemokine, in osteoarthritis. Osteoarthritis Cartilage.

[34] Mocellin S, Panelli MC, Wang E, Nagorsen D, Marincola FM (2004). The Dual Role of IL10. Trends in Immunology.

[35] Moore KW, Malefyt R, Coffman RL, O’Garra A (2001). Interluekin-10 and the interluekin-10 receptor. Annual Review of Immunology.

[36] Kermanizadeh A (2017). Nanomaterial-induced cell death in pulmonary and hepatic cells following exposure to three different metallic materials: The role of autophagy and apoptosis. Nanotoxicology.

[37] Gosens I (2015). Comparative hazard identification by a single dose lung exposure of zinc oxide and silver nanomaterials in mice. Plos One.

[38] Kermanizadeh A, Gaiser BK, Hutchison GR, Stone V (2012). An *in vitro* liver model - assessing oxidative stress and genotoxicity following exposure of hepatocytes to a panel of engineered nanoparticles. Particle and Fibre Toxicology.

[39] Kermanizadeh A, Gaiser BK, Ward MB, Stone V (2013). Primary human hepatocytes vs. hepatic cell line - assessing their suitability for *in vitro* nanotoxicology. Nanotoxicology.

[40] Kermanizadeh A, Jacobsen NR, Roursgaard M, Loft S, Møller P (2017). Hepatic hazard assessment of silver nanoparticle exposure in healthy and chronically alcohol fed mice. Toxicological Sciences.

[41] Nguyen-Lefebvre AT, Horuzsko A (2015). Kupffer cell metabolism and function. Journal of Enzymology and Metabolism.

[42] Beljaars L (2014). Hepatic localization of macrophage phenotypes during fibrogenesis and resolution of fibrosis in mice and humans. Frontiers in Immunology.

[43] Aalapati S, Ganapathy S, Manapuram S, Anumolu G, Prakya BM (2014). Toxicity and bio-accumulation of inhaled cerium oxide nanoparticles in CD1 mice. Nanotoxicology.

[44] Shrivastava R, Raza S, Yadav A, Kushwaha P, Flora SJS (2014). Effects of sub-acute exposure to TiO_2_, ZnO and Al_2_O_3_ nanoparticles on oxidative stress and histological changes in mouse liver and brain. Drug and Chemical Toxicology.

[45] Bell CC (2018). Comparison of hepatic 2D sandwich cultures and 3D spheroids for long-term toxicity applications: a multicentre study. Toxicological Sciences.

[46] Sahi J, Grepper S, Smith C (2010). Hepatocytes as a tool in drug metabolism, transport and safety evaluations in drug discovery. Current Drug Discovery Technologies.

[47] Baffy G (2009). Kupffer cells in non-alcoholic fatty liver disease: the emerging view. Journal of Hepatology.

